# Connectivity of Random Geometric Hypergraphs

**DOI:** 10.3390/e25111555

**Published:** 2023-11-17

**Authors:** Henry-Louis de Kergorlay, Desmond J. Higham

**Affiliations:** School of Mathematics, University of Edinburgh, Edinburgh EH9 3FD, UK; hdekerg@ed.ac.uk

**Keywords:** bipartite, radius, random graph

## Abstract

We consider a random geometric hypergraph model based on an underlying bipartite graph. Nodes and hyperedges are sampled uniformly in a domain, and a node is assigned to those hyperedges that lie within a certain radius. From a modelling perspective, we explain how the model captures higher-order connections that arise in real data sets. Our main contribution is to study the connectivity properties of the model. In an asymptotic limit where the number of nodes and hyperedges grow in tandem, we give a condition on the radius that guarantees connectivity.

## 1. Motivation

There is growing interest in the development of models and algorithms that capture group-level interactions [1,2,3]. For example, multiple co-authors may be involved in a collaboration, multiple workers may share an office space, and multiple proteins may contribute in a cellular process. In such cases, representing the connectivity via a network of pairwise interactions is an obvious, and often avoidable, simplification. Hypergraphs, where any number of nodes may be grouped together to form a hyperedge, form a natural generalisation. Hypergraph-based techniques have been developed for the following:

- Studying the propagation of disease or information [4,5,6,7,8,9];
- Investigating the importance or structural roles of individual components [10,11,12];
- Discovering and quantifying clusters [13,14,15];
- Predicting future connections [16,17];
- Inferring a connectivity structure from time-series data [18].

Just as in the pairwise setting, it is also of interest to consider processes that create hypergraphs [19,20,21]. Comparing generative hypergraph models against real data sets may help us to understand the mechanisms through which interactions arise. Furthermore, realistic models can be used to produce synthetic data sets on which to base simulations and also to form null models for studying features of interest.

Models that use a geometric construction, with connectivity between elements determined by distance, have proved useful in many settings. Random geometric graphs were first introduced in [22] to model communication between radio stations, although the author also mentioned their relevance to the spread of disease. These models have subsequently proved useful in many application areas, ranging from studies of the proteome [23,24,25] to academic citations [26]. In many settings, the notion of distance may relate to the embedding of nodes into a latent space that captures key features. Here, similarity is interpreted in an indirect or abstract sense. Random geometric graphs have also been studied theoretically, with many interesting results arising from the perspectives of analysis, probability, and statistical physics [27,28,29,30,31,32].

Our aim in this work is to motivate and analyse a random geometric hypergraph model. In a similar manner to [19], we make use of the connection between hypergraphs and bipartite graphs. The model is introduced and motivated in Section 2, where we also show the results of illustrative computational experiments concerning connectivity. Our main contribution is to derive a condition on the thresholding radius that asymptotically guarantees connectivity of the hypergraph. The result is stated and proved in Section 3. Some further computations concerning the expected degree are presented and interpreted in Section 4, and directions for future work are described in Section 5.

## 2. The Random Geometric Model and Its Connectivity

In this section, we motivate and informally describe a random geometric hypergraph model and computationally investigate its connectivity. We make use of a well-known equivalence between hypergraphs and bipartite graphs [19,33]. Suppose we are given an undirected bipartite graph where nodes have been separated into two groups, A and B. By construction, any edge must join one node in group A with one node in group B. We may form a hypergraph on the nodes in group A with the following rule:

- Nodes in group A appear in the same hyperedge if and only if, in the underlying bipartite graph, they both have an edge to the same node in group B.

In this way, the nodes in set B may be viewed as hyperedge “centres.” Two nodes from group A that are attracted to the same centre are allocated to the same hyperedge. In many graph settings, there is a natural concept of distance between nodes. For example, in social networks, geographical distance between places of work or residences may play a strong role in determining connectivity. More generally, there may be a more nuanced set of features (hobbies, tastes in music, pet ownership, etc.) that help to explain whether pairwise relationships arise. This argument extends readily to the bipartite/hypergraph scenario. Hyperedge centres may correspond, for example, to shops, office buildings, gyms, train stations, restaurants, concert venues, churches, etc., with an individual joining a hyperedge if they are sufficiently close to that centre, for example, exercising at a local gym. In the absence of specific information, it is natural to assume that the features possessed by a node arise at random, so that a node is randomly embedded in $R^{d}$ for some dimension *d*. In a similar way, we may simultaneously embed our hyperedge centres in $R^{d}$ and assign a node to a hyperedge if and only if it is within some threshold distance of the centre.

**Figure 1:**
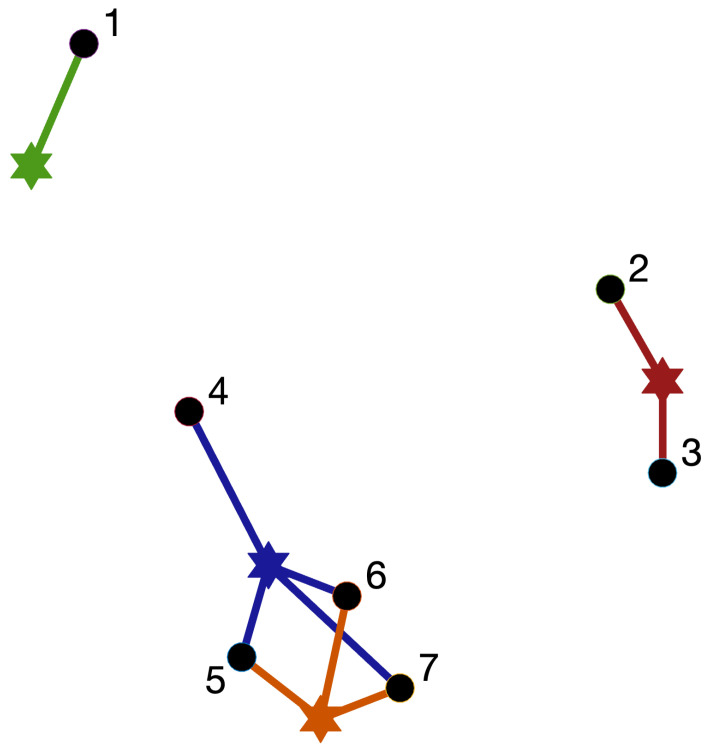
When this construction is regarded as a bipartite graph, the solid circles and solid stars represent two types of nodes. Edges are created only between nodes of a different type; this happens if and only if they are close enough in Euclidean distance. When regarded as a hypergraph, the solid circles represent nodes and the solid stars represent “centres” of hyperedges. A node is a member of a hyperedge if and only if it is sufficiently close to the corresponding centre. Mathematically, the resulting hypergraph may be defined by labelling the nodes {1,2,3,4,5,6,7} and listing the hyperedges as {1}, {2,3}, {4,5,6,7}, and {5,6,7}.

Figure 1 illustrates the idea in the two-dimensional case. We have a bipartite graph with two types of nodes. Groups A and B are represented by circles and stars, respectively. We form a hypergraph by placing a circle node in a hyperedge if and only if it is within a certain distance of the corresponding star. Colours in the figure distinguish between the different hyperedges. We emphasise that mathematically the resulting hypergraph consists only of the list of hypergraph nodes and hyperedges. Information about the existence/number of hyperedge centres and the locations of all nodes in $R^{2}$ is lost.

Our aim in this work is to study connectivity: a basic property that is of practical importance in many areas, including disease propagation, communication, and percolation. We consider the random geometric hypergraph to be connected if the underlying random geometric bipartite graph is connected. We focus on the smallest distance threshold that produces a connected network and study an asymptotic limit where the number of nodes tends to infinity.

**Figure 2:**
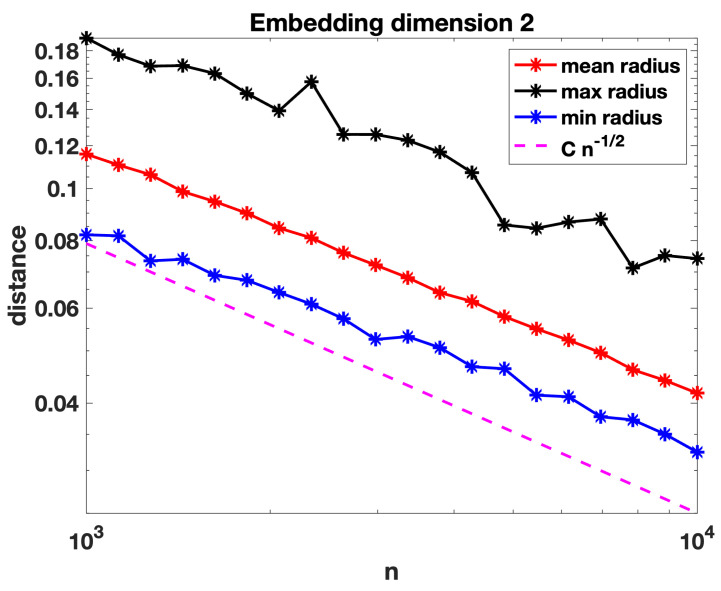
Euclidean distance at which random geometric hypergraph becomes connected. Here, we have 0.8n nodes and 0.2n hyperedge centres in $R^{2}$, for values of *n* between $10^{3}$ and $10^{4}$. The plots show the mean, maximum, and minimum value of this distance over 500 independent trials. A reference slope corresponding to $Cn^{-\frac{1}{2}}$ is shown. Axes are logarithmically scaled. Largest standard error for the mean computations was below $10^{-3}$.

We motivate our analytical results with computational experiments. To produce Figure 2, we formed random geometric bipartite graphs based on *n* points embedded in $R^{2}$. For each graph, the points had components chosen uniformly and independently in the range (0,1). We separated these points into two groups of size $n_{1}=0.8n$ and $n_{2}=0.2n$. We then used a bisection algorithm to compute the smallest radius *r* that produced a connected bipartite graph. In other words, we found the smallest *r* such that a connected graph arose when we created edges between pairs of nodes from different groups that were separated by a Euclidean distance less than *r*. (Equivalently, we assigned $n_{1}=0.8n$ points to the role of the nodes in a random geometric hypergraph and $n_{2}=0.2n$ points to the role of the hyperedge centres, and we computed the smallest node–hyperedge centre radius that gave connectivity.) We ran the experiment for a range of *n* values between $10^{3}$ and $10^{4}$. For each choice of *n*, we repeated the computation for 500 independent random node embeddings. Figure 2 shows the mean, maximum, and minimum radius arising for each *n*. Note that the axes are scaled logarithmically. We have superimposed a reference line of the form $Cn^{-\frac{1}{2}}$, which is seen to be consistent with the behaviour of the radius.

**Figure 3:**
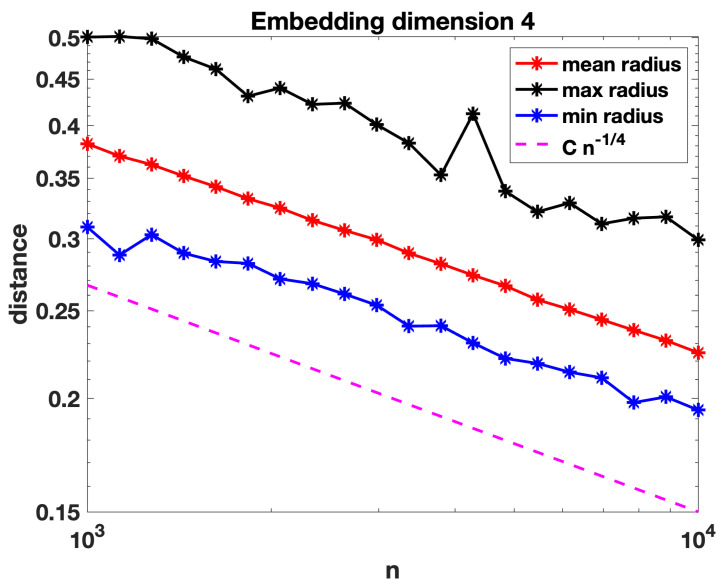
As for Figure 2, with nodes embedded in $R^{4}$ and a reference slope corresponding to $Cn^{-\frac{1}{4}}$. Largest standard error for the mean computations was below $10^{-2}$.

**Figure 4:**
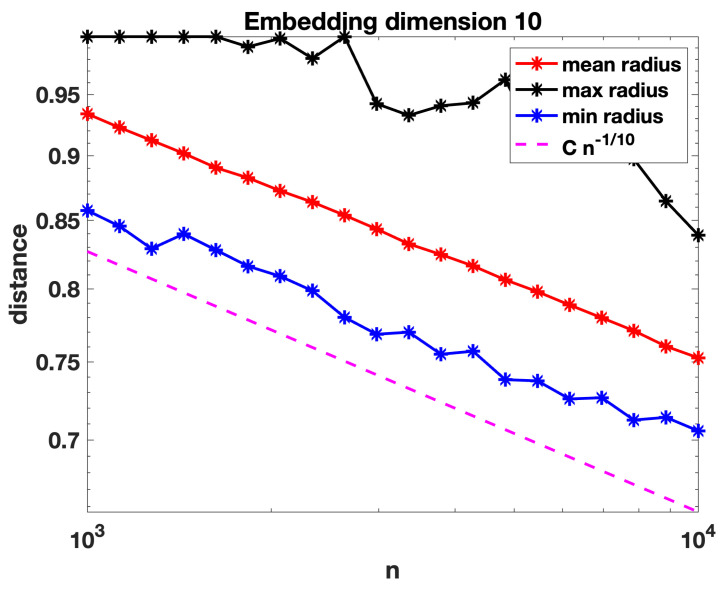
As for Figure 2, with nodes embedded in $R^{10}$ and a reference slope corresponding to $Cn^{-\frac{1}{10}}$. Largest standard error for the mean computations was below $10^{-2}$.

Figure 3 and Figure 4 repeat these computations with the points embedded into $R^{4}$ and $R^{10}$, respectively. We see that the behaviour remains consistent with a decay roughly proportional to, and perhaps slightly slower than, $n^{-1/d}$ for dimension *d*.

In the next section, we formalise our definition of a random geometric hypergraph and establish a condition on the radius decay rate for connectivity that agrees with $n^{-1/d}$, up to log-dependent factors (which of course would be extremely difficult to pin down in computational experiments). We also note for comparison that a threshold of the form ${\left(\log\left(n\right)/n\right)}^{1/d}$ has previously arisen in the study of random geometric graphs, refs. [30,34].

In related work, we note that Barthelemy [19] proposed and studied a wide class of random hypergraph models, including examples where nodes are embedded in space and connections arise via a distance measure. That approach to defining a random geometric hypergraph differs from ours by assuming that the number of hyperedges is given and by considering a process where new nodes are added to the network, with new connections arising based on the current hyperedge memberships (Figure 6 in [19]).

## 3. Connectivity Analysis

We now give a formal definition of a random geometric hypergraph and show that under reasonable conditions a thresholding radius of order ${\left(\log\left(n\right)/n\right)}^{1/d}$ ensures connectivity, asymptotically.

Let *D* be a bounded Euclidean domain in $R^{d}$ such that *D* has a Lipschitz boundary. Given n∈N, we let $P_{n}$ be a Poisson point process sampled from *D* with respect to some continuous and bounded distribution *f* such that f>0 everywhere on *D*. We use |·| to denote the Euclidean norm. Let n∈N, and let $n_{1}$ be the expeted number of nodes and $n_{2}$ be the expected number of hyperedges, chosen such that $n=n_{1}+n_{2}$. Let $r_{n}$ be a function of *n*, tending to 0 as n→∞.

**Definition** **1.** *Let $G(n_{1},n_{2},r_{n})$ be the probability space on the set of geometric hypergraphs, where the random nodes are chosen as a Poisson point process $P_{n_{1}}$ in D sampled with respect to f; the random hyperedges are induced by another Poisson point process $P_{n_{2}}$ in D sampled with respect to f; and where, using bipartite graph–hypergraph equivalence, a node $x\in P_{n_{1}}$ and a hyperedge $y\in P_{n_{2}}$ are connected by an edge if $|x-y|<r_{n}$.*

Suppose that the expected number of nodes $n_{1}$ and of hyperedges $n_{2}$ satisfy

$$\frac{n_{1}}{n_{2}}=\Theta \left(1\right).$$

Equivalently, this means that $n_{1}$ and $n_{2}$ as functions of *n* satisfy

$$n_{1}=\Theta \left(n\right),\;n_{2}=\Theta \left(n\right).$$

Let K>0 be the smallest constant such that for all n∈N,

(1) $$n_{1}\geq \frac{1}{K}n\;\;\mathrm{and}\;\;n_{2}\geq \frac{1}{K}n.$$

Partition $R^{d}$ into a grid of cubes ${\left\{C_{i,n}\right\}}_{i}$ of width $\gamma r_{n}$, where $r_{n}=o\left(1\right)$ and γ>0 is to be determined. Let $S_{n}:=\left\{i\;|\;C_{i,n}\subset D\right\}$, and for each $i\in S_{n}$, let $I\left(i,n\right):=\left\{j\notin S_{n}\;|\;C_{j,n}\;\mathrm{is}\;\mathrm{adjacent}\;\mathrm{to}\;C_{i,n}\right\},$ and let

$$Q_{i,n}:=\cup_{j\in I(i,n)}\left(C_{j,n}\cap D\right).$$

Because *D* has a Lipschitz boundary, by compactness there exists C>0 depending on *D* and *d* (but not on γ), such that we can choose $n_{0}\in N$ sufficiently large such that for all $n\geq n_{0}$ and all $i\in S_{n}$

$$\forall \;x,y\in Q_{i,n},\;\left|x-y\right|<C\gamma r_{n}.$$

We then choose $\gamma :=\frac{1}{C},$ so that for all $i\in S_{n}$,

(2) $$\forall \;x,y\in Q_{i,n},\;\left|x-y\right|<r_{n}.$$

Note also that we have $\nu \left(Q_{i,n}\right)\geq \nu \left(C_{i,n}\right)\geq f_{\min}\gamma^{d}r_{n}^{d}=\frac{f_{\min}}{C^{d}}r_{n}^{d},$ where $f_{\min}:=\min\left\{f\left(x\right)\;|\;x\in \Omega \right\}.$

**Lemma** **1** (Asymptotic coverage). *Suppose that m as a function of n satisfies, for all n∈N,*

$$m\geq \frac{1}{K}n,$$

*and suppose that $r_{n}$ satisfies*

(3) $$n\frac{f_{\min}}{KC^{d}}r_{n}^{d}\geq \log n-\log\log n+w\left(n\right),$$

*where w(n)→∞ arbitrarily slowly as n→∞. With the probability tending to *1* as n→∞, for all $i\in S_{n}$,*

$$P_{m}\cap Q_{i,n}\neq \emptyset .$$

**Proof.**  It suffices to show that the RHS in

$$P\left(\exists \;i\in S_{n},P_{m}\left(Q_{i,n}\right)=0\right)\leq \sum_{i\in S_{n}}P\left(P_{n}\left(Q_{i,n}\right)=0\right)$$

tends to 0 as n→∞.

Because $P_{m}$ is a homogeneous Poisson point process, we have, using (3), for all $i\in S_{n}$,

$$P\left(P_{m}\left(Q_{i,n}\right)=0\right)=\exp\left(-m\nu \left(Q_{i,n}\right)\right)\leq \exp\left(-n\frac{f_{\min}}{KC^{d}}r_{n}^{d}\right)\leq n^{-1}\left(\log n\right)e^{-w(n)},$$

and by the pigeonhole principle, $|S_{n}|≲{\left(\gamma r_{n}\right)}^{-d}≲n{\left(\log n\right)}^{-1}$. Hence,

$$\begin{array}{cc} P(\exists \;i\in S_{n},P_{n}\left(Q_{i,n}\right)=0) & ≲e^{-w(n)}. \end{array}$$

□

Note that with our choice of γ, we have

$$\left\{\forall \;i\in S_{n}\;|\;P_{m}\cap Q_{i,n}\neq Ø\right\}\subset \left\{D\subset \cup_{x\in P_{m}}B\left(x,r_{n}\right)\right\}.$$

Hence, Lemma 1 gives us a lower-bound estimate on the decay of $r_{n}$ as a function of *n*, to ensure that the balls centred at the points of $P_{m}$ and of radius $r_{n}$ tend to form a covering of the domain *D* as n→∞. This is an asymptotic result.

From a practical point of view, it is more useful to have a non-asymptotic version of Lemma 1, even if we must increase slightly the constraint on the decay of $r_{n}$. This is the object of Lemma 2.

**Lemma** **2** (Non-asymptotic coverage). *Suppose that m as a function of n satisfies, for all n∈N,*

$$m\geq \frac{1}{K}n,$$

*and suppose this time that $r_{n}$ satisfies*

(4) $$n\frac{f_{\min}}{KC^{d}}r_{n}^{d}\geq 2\log n+\epsilon \log\log n,$$

*for some fixed ϵ>0, then a.s., there exists N∈N such that for all n≥N and all $i\in S_{n}$*

$$P_{m}\cap Q_{i,n}\neq Ø.$$

**Proof.**  A proof proceeds similarly to that of Lemma 1, but the different constraint on $r_{n}$ instead yields

$$P\left(\exists \;i\in S_{n},P_{m}\left(Q_{i,n}\right)=0\right)≲\frac{1}{n{\left(\log n\right)}^{1+\epsilon }},$$

and the required result then follows by using the Borel–Cantelli lemma, because then, the series

$$\sum_{n=0}^{N}\;P\left(\exists \;i\in S_{n},P_{m}\left(Q_{i,n}\right)=0\right)$$

converges as N→∞. □

We believe that the lower-bound condition on the decay of $r_{n}$ found in Lemma 1 is sharp and that the lower-bound condition in Lemma 2 is close to being sharp. In Theorem 1, we apply Lemmas 1 and 2 to obtain a sufficient lower-bound condition on $r_{n}$ for the connectivity of random geometric hypergraphs, with an extra factor of 2. We suspect that this factor could be reduced with a more sophisticated analysis.

**Theorem** **1.** *For every n∈N, let $\left(n_{1},n_{2}\right)\in N^{2}$ satisfy (1) and $n=n_{1}+n_{2}$.*

- *If $r_{n}$ satisfies (3), then with the probability tending to *1* as n→∞, the random geometric bipartite graph $G(n_{1},n_{2},2r_{n})$ is connected.*

- *If $r_{n}$ satisfies (4), then a.s. there exists N∈N, such that for all n≥N, the random geometric bipartite graph $G(n_{1},n_{2},2r_{n})$ is connected.*

**Proof.**  The result is a consequence of Lemmas 1 and 2 and the triangle inequality.

Suppose that n∈N is such that for all $i\in S_{n}$,

(5) $$P_{n_{1}}\cap Q_{i,n}\neq Ø\;\;\mathrm{and}\;\;P_{n_{2}}\cap Q_{i,n}\neq Ø.$$

Given two points $x,y\in P_{n_{1}}$, we can find a path of adjacent cubes from $Q_{n}$ such that the first cube contains *x* and the last cube contains *y*. From (2) and the triangle inequality, the distance between a point in one cube and another point in an adjacent cube is at most $2r_{n}$. Because for each cube in the path we can find a point from $P_{n_{1}}$ and a point from $P_{n_{2}}$, we can then form a path of edges of a length that is at most $2r_{n}$ from *x* to *y*, alternating between points in $P_{n_{1}}$ and points in $P_{n_{2}}$, and such a path is then a path in $G(n_{1},n_{2},2r_{n}).$

This shows the connectivity of the graph for all *n*, satisfying condition (5).

This condition is true with the probability tending to 1 as n→∞, if we assume that $r_{n}$ satisfies (3), using Lemma 1 with $n_{1}$ and $n_{2}$ instead of *m*, giving us the first part of the theorem.

Using Lemma 2 with $n_{1}$ and $n_{2}$ instead of *m*, if $r_{n}$ satisfies (4), there exists N∈N such that (5) is true for all n≥N, giving us the second part of the theorem. □

## 4. Expected Degree at Connectivity Threshold

**Figure 5:**
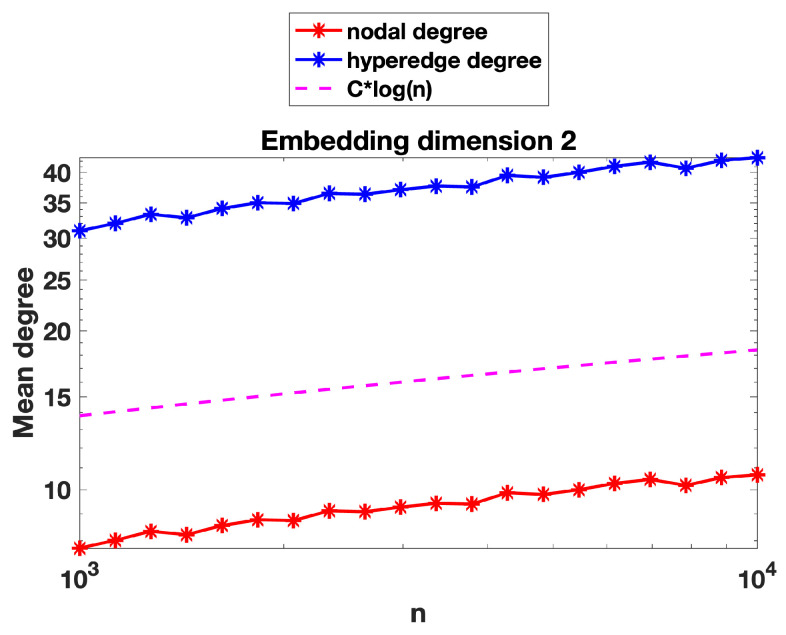
For the geometric random hypergraphs in Figure 2, we show the mean node degree and mean hyperedge degree. A reference slope corresponding to Clogn is also plotted. Axes are logarithmically scaled. Largest standard error for the mean computations was below 0.2 for nodal degree and below 0.6 for hyperedge degree.

**Figure 6:**
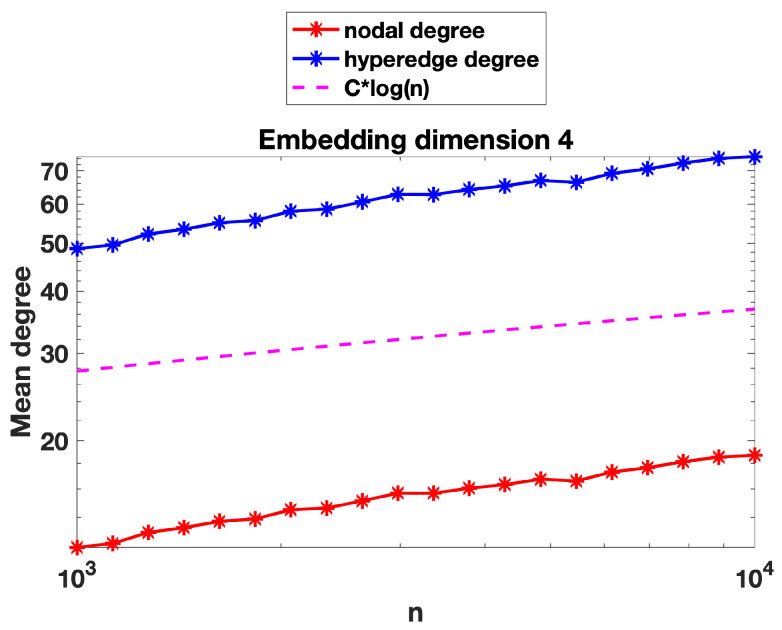
As for Figure 5, with nodes embedded in $R^{4}$. Largest standard error for the mean computations was below 0.3 for nodal degree and below 1 for hyperedge degree.

**Figure 7:**
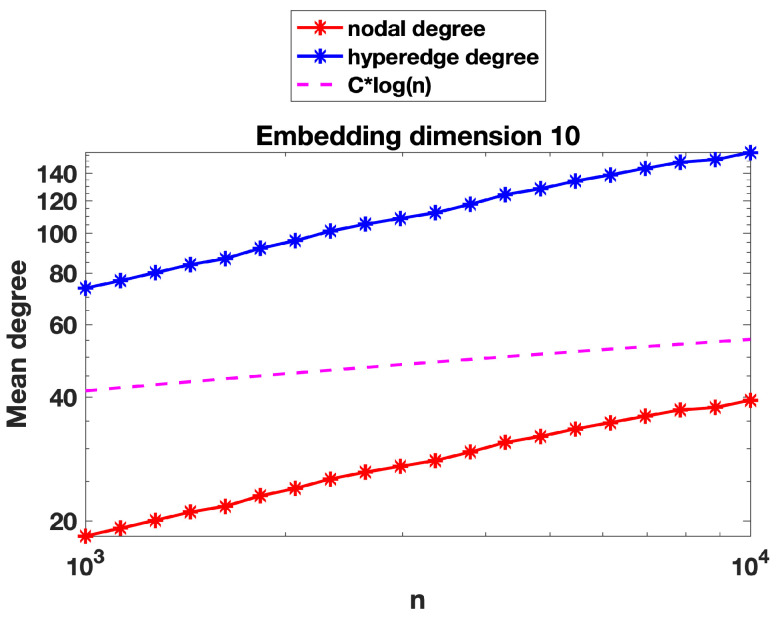
As for Figure 5, with nodes embedded in $R^{10}$. Largest standard error for the mean computations was below 0.5 for nodal degree and below 2 for hyperedge degree.

We now present some further computations that expand on the results in Section 3. We recall that in Figure 2, Figure 3 and Figure 4 we evaluated the threshold radius at which connectivity occurred. In Figure 5, Figure 6 and Figure 7 we used the same random geometric hypergraph samples, each evaluated at its connectivity threshold. For each hypergraph, we computed the expected node degree and expected hyperedge degree, that is, for the underlying bipartite graph, the expected degree of the nodes in group A and in group B. The figures, which again are on a log–log scale, indicate that the two expected degrees grow slowly with *n*. In each figure, we have included a reference curve proportional to log(n).

We can offer a heuristic explanation for these curves. To be concrete, we focus on the nodes in group A of the bipartite graph. Here, because the group B nodes are placed uniformly at random, the expected degree is roughly the number of group A nodes contained in a general ball of radius *r*, where *r* is the connectivity radius. We can compute this quantity as the sum of the independent probabilities that each node is in the ball, which is of the order of the volume of the ball, that is, $r^{d}$. Because there are order *n* nodes, this suggests an expected degree of the order $nr^{d}$. Using $r∼{\left(\log n/n\right)}^{1/d}$ from Section 3 for the threshold radius, we arrive at an expected degree of order logn. This prediction gives a reasonable match to the results in Figure 5 and Figure 6, where the embedding dimensions are two and four, respectively.

However, we note that in Figure 7, where the embedding dimension is ten, the mean node degree and hyperedge degree appear to grow slightly faster than Clog(n). We believe that in a high dimension, a concentration-of-measure effect becomes relevant. In order to have more meaningful information on the mean degree, we would like to be able to control simultaneously the number of nodes of graph A in the *n* balls of radius *r* centred at the *n* nodes of graph B and to be able to argue that this random number behaves asymptotically like *n* times the volume of the ball (the expected number), i.e., remains near its expected value for each of the *n* balls. For this to be true, we would need some concentration inequalities, which would ensure that the empirical measure induced by the random sample of the nodes of graph A yields a good approximation of the underlying sampling measure when evaluated at *n* random balls of radius *r*. Such concentration inequalities are known to hold in a regime slightly more restrictive than that of connectivity, i.e., where $nr^{d}$ grows slightly faster than Clog(n); see, for instance, Lemma 3.2 in [35], where such concentration inequalities are valid provided $nr^{d}=\omega \left(\log n\right)$.

A second issue is that the effect of interchanging the order in which the expectation operation and limn→∞ operation are applied cannot be understood without careful analysis.

We leave for future work the task of formalising and proving appropriate asymptotic statements about the degree structure for this random geometric hypergraph model.

## 5. Discussion

There are a number of promising avenues for further work in this area. From a theoretical perspective, it would be of interest to derive sharper upper and lower bounds, or indeed exact expressions, for the connectivity radius threshold associated with this class of random geometric hypergraphs. More general hypergraph models could also be developed and studied, for example, using a softer version of the distance cut-off that has been considered in the graph setting [31,32], and other properties of the model could be investigated.

From a more practical viewpoint, the related inverse problem is both challenging and potentially useful: given a data set that corresponds to a hypergraph, for the model considered here, what is the best choice of (a) embedding dimension, (b) node locations, and (c) hypergraph centre locations? A similar question was addressed in [21] for a different generative random hypergraph model based on the assumption that nodes are located in a latent space and hyperedges arise preferentially between nearby nodes (without the concept of hyperedge centres). This challenge also leads into the model selection question: given a data set and a collection of hypergraph models, which model best describes the data, and what insights arise? 

## Data Availability

Code for the experiments reported here may be found at https://www.maths.ed.ac.uk/dhigham/algfiles.html.
